# Competency-based Training Needs Assessment for Research Managers and Administrators in Africa and the United Kingdom to Strengthen Equitable Partnerships

**DOI:** 10.12688/aasopenres.13272.1

**Published:** 2022-03-10

**Authors:** Victoria Nembaware, Simon Glasser, Anne Priest, Ailsa Davies, Michelle Skelton, Paidamoyo Bodzo, Olivia Lelong, Alecia Naidu, Colleen Masimirembwa, Alice Mutambiranwa, Annette Hay, Ambroise Wonkam, Collet Dandara

**Affiliations:** 1 Division of Human Genetics, Department of Pathology, Faculty of Health Sciences, University of Cape Town, Cape Town,, 7925, South Africa; 2 Research and Enterprise Development, University of Bristol, Bristol, BS8 1QU, UK; 3 Research and Innovation Services, University of Bath, Bath, BA2 7AY, UK; 4 Research and Innovation Services,, Cardiff University, Cardiff, CF10 3AT, UK; 5 H3Africa Administrative Coordinating Centre, Integrative Biomedical Sciences, Faculty of Health Sciences, University of Cape Town, Cape Town, 7925, South Africa; 6 Research, Business & Innovation Service, University of the West of England, Bristol, BS16 1GY, UK; 7 South African National Bioinformatics Institute, University of the Western Cape, Cape Town, 7535, South Africa; 8 African Institute of Biomedical Science and Technology (AiBST), Harare, Zimbabwe; 9 Research Services Office, Coventry University, Coventry, CV1 5FB, UK

**Keywords:** Research Manager and administrators, Africa, United Kingdom, Competencies, Needs Assessment

## Abstract

**Background:** The need for competent research managers and administrators (RMAs) has increased due to the complexity in managing research projects between disparate and international partners. To facilitate the creation of robust training and professional development programmes it is essential to first understand the status quo. A collaborative project, Sustainable Management and Administration for Research: Training across the project Lifecycle (SMARTLife), made up of RMAs from South Africa, Zimbabwe and the United Kingdom (UK) developed a set of competencies to conduct an RMA competency-based training needs assessment scoping tool.

**Method:** Nine areas were identified: Equitable partnership; Finance Management; Project Management; Monitoring and Evaluation; Reporting and Communications; Equity, Diversity & Inclusion; Training and Capacity Development; Impact a& Sustainability; and Ethical, Social, Legal a& Social Implications.  Tasks for each competency area were identified to develop an scoping tool that had 168 data collection points. The tool was advertised through press releases, mailing lists and social media.

**Results:**  108 responses were obtained:  with 49% from 15 Africa countries/the remainder from the UK. The UK (71%) had more permanent RMA staff members compared to Africa (39%). There were more respondents in Africa with the title of Research Manager/Coordinator(p=0.0132) compared to the UK where most of the RMAs were employed as Finance/Contract officers. 60% of respondents from the UK had more than three years experience while only 35% from Africa had experience. While most RMAs had formal higher education qualifications, their training was not in research management and administration, which requires a diverse range of skills. Confidence in specific tasks varied between the UK and Africa whereas collaborative partnerships challenges and enablers were similar.

**Conclusion** This work highlights differences in RMA training and experience RMA  between Africa and UK, this work could inform much needed competency-based training for RMAs and partnership strategies that aid mutual-learning.

## Introduction

Research managers and administrators (RMAs) support research projects throughout the project lifecycle. They are key to effective research governance often taking up the bulk of the administrative burden away from academics and researchers ( Langley & Green, 2009; Tauginienė, 2009). Increased need for tighter funder reporting requirements, complex institutional procedures and policies especially in large-scale collaborative research projects have created a need for dedicated and trained RMAs. The roles and tasks of RMAs vary greatly from providing support to specific projects, to faculty and even institutional level support, across the entire project cycle or at specific points ( Langley & Green, 2009; Tauginienė, 2009). In addition, the roles and responsibilities of RMAS are continually evolving to match the ever-changing research cultures and infrastructures. For example, traditionally, research was conducted in small teams but the research culture is fast changing with the growing needs of multi-national collaborations. However, unlike developed regions such as the United States of America (USA) and the United Kingdom (UK) many research institutions in Africa are yet to establish dedicated RMA officers and processes ( Akindele & Kerridge, 2019). Research capacity development activities, irrespective of region, have largely disregarded the upskilling of RMAS and focused mainly on academics or specific research support technical professionals such as laboratory technicians and data managers ( Bennett *et al*., 2013; Karikari *et al*., 2015).

The general global-wide disregard of the need to strengthen RMAs skills and competencies is highlighted by the limited trainings provided by academic institutes ( Table 1). As highlighted in Table 1, existing training programmes are mainly available in the north and therefore are likely to lack content that addresses need from the global south. Some of the programmes which do address skills needs from developing countries are subject-specific, such as Global Health and may also be limited in applicability to RMAs in academic or research institutes if they focus on case studies from not-for-profit organizations ( University of Washington, 2009).

**Table 1.** Existing Training Programmes Relevant to Research and Administration field.

Level of training	Institute	Country, Continent	Title of Course/Training (link)	Comments
Certificate	Washington University	USA	https://edgh.washington.edu/courses/project-management?utm_ source=eDGH+Master&utm_campaign=8bc3bdda1e-EMAIL_CAMPAIGN_2020_04_01_11_ 40_COPY_01&utm_medium=email&utm_term=0_a7e169cc45-8bc3bdda1e-455369562	Project management in Global Health. Cases studies and lectures all geared towards managing projects and research within "Not for profit" organizations.
Certificate	Emmanuel College Boston	USA	https://www.emmanuel.edu/graduate-and-professional-programs/business-and- management/research-administration.html	Established course
Post-graduate diploma	Stellenbosch University	South Africa, Africa	https://www0.sun.ac.za/crest/news/post-graduate-diploma-in-research-management- and-administration/	Currently the only University- based training in Africa specific for RMAs
Master of Science	John Hopkins	Washington, USA	https://advanced.jhu.edu/academics/graduate-degreeprograms/research- administration-4/research-administration-4/	Online training
Masters of Research Administration and Certification	University of Florida	Florida, USA	https://www.ucf.edu/online/research-administration/	Online training
Certificate in Research Management	ARMA	United Kingdom	https://arma.ac.uk/	Fee to join and fee to attend courses. Wide range of course topics
Certificate	SARIMA	Southern Africa	https://www.sarima.co.za/events/#01	Online training

NB: Level of training= **PhD, MSc, Bachelor, Diploma, Certificate**

Most existing training and professional development opportunities ( Table 1) we identified are available through RMAs professional associations such as the Association of Research Managers and Administrators (ARMA), UK and the European Association of Research Managers and Administrators (EARMA), but are mostly limited to their respective members and also very costly ( ARMA, 2021; EARMA, 2021). Training available to non-members might also have conditions which for example do not allow RMAs without experience to participate ( “EARMA Certificate in Research Management >> WBC-RTI.INFO - Western Balkan Countries Research Technology Innovation,” 2018). In Africa, the Southern Africa Research and Innovation Management Association (SARIMA) also offers short online courses on research management and while it is open to non-members, it is also relatively expensive. The West African Research and Innovation Management Association (WARIMA) regularly organises training workshops for its members while trinity facilitate training of RMAs. These different training activities could partner with academic institutes which to make the courses more affordable and accessible to persons in academia who are involved, or an interested in a career in research management and administration.

A number of training needs assessments in RMA field have been conducted, ( Langley & Green, 2009; Tauginienė, 2009; Virágh *et al*., 2019). However, these assessments have some limitations. Of note is a needs assessment conducted as a business case for the ERAMUS project through a mixed-method approach ( Langley & Barsby, 2020). While this needs assessment captured valuable implementation information, it is biased towards the Southern African due to funding stipulations. Given, the trend towards internationalization of research, such a needs assessment missed an opportunity to critically assess differences in responses between different geographical regions as such differences have potential to highlight issues that could negatively impact equitable international research partnerships. The creation of any professional training for RMAs needs to be broad enough to cater for a vast range of competencies required while catering for diverse and sometimes disparate partnerships.

Competence-based needs assessments are widely used across different areas to inform design of training programmes ( Frank *et al*., 2010; Kim & Roh, 2019; Mulder *et al*., 2018; Nembaware *et al*., 2019). Such needs assessments commonly use a set of competencies to capture existing roles as well as assess the current training needs and gaps. To identify training needs to RMAs in the UK and Africa, we established The Sustainable Management and Administration for Research: Training across the project Lifecycle (SMARTLife), a collaborative project between RMAs from Southern Africa and the UK, we developed, a) a set of competences for RMAs and b) used these competencies to conduct an RMAs skills and training needs assessment for international collaborations through a comparative assessment of participants from both the UK and Africa.

## Method

The competence areas were compiled from the ARMA webpage and adjustments were made by SMARTLife members to make these more relevant to their current experiences through group discussions. The SMARTLife team developed a scoping tool which had three main sections ( SMARTLife, 2021b):

1. **Demographic questions** name, age range, location, gender, highest level of education, current role, type of employer, years of experience, training received for RMA role, tasks participant perform in their current roles.
2. **Levels of confidence in specific competence areas:** Current responsibilities and how confident participants feel undertaking certain tasks relating to international research partnerships; the main challenges associated with international partnerships; approaches that have worked well and the available resources.
3. **Aspects related to future collaborations with SMARTLife**: How survey participants may like to engage with future training activities, to promote knowledge exchange amongst.

The scoping tool was developed in the REDCap system. Piloting and adjustments of the tool were conducted by the SMARTLife team members and with a few selected external RMAs participants. The three sections were further broken down into 9 competencies areas as listed below:

a) **Equitable partnerships:** Sustainable research collaborations depend on, among other things, fair research partnerships, co-ownership, capacity development, and their impact on improving social and academic outcomes. As part of the project, the team evaluated several tasks that gauge equitable partnerships and these included looking at how trust within partnerships is built, facilitated, as well as sustained. In this section, the project investigated how partnering teams (i) navigate funding obligations taking into account differing local contexts, (ii) contribute to a fair working environment taking into account of cultural differences, (iii) promote or support mutual learning, (iv) evaluations that take into account differing needs of target audiences (e.g. project beneficiaries, funders etc) in order to improve collaborations.
b) **Financial Management:** Financial management is fundamental to securing funding, managing projects, balancing budgets, handling operations, ensuring compliance and providing support in many other capacities. Major areas of consideration in financial management which informed the questions posed to participants include the development of budgets; ensuring adherence to funders terms and conditions and procuring what is needed for the project; maintaining the staff working on the project.
c) **Project management:** Critical tasks that informed questions asked in this area include preparation of bids between international partners; establishing project plans or policies; creating project management platforms; coordination and communication; achieving project deliverables; building or maintaining relationships with funders, partners or other stakeholders; collecting and collating data and project reporting to funders and other key stakeholders.
d) **Monitoring and Evaluation (M&E):** Questions on M&E in the scoping survey touched on designing of M&E frameworks and indicators; conducting due diligence of partners or sub-awardees; collecting monitoring data; conducting data analyses; and writing evaluation reports.
e) **Reporting and Communications:** Communication covers aspects within teams, between teams, and across to funders and the general public. Important tasks in this section included: designing and implementing communication plans; engaging with the media; developing funder reports and all engagements with key stakeholders.
f) **Equality, Diversity, and Inclusion:** Diversity can be an asset in collaborative teams or partnerships however, it mostly works when this diversity is tapped into, with equality in mind, thus enriching a project through merging of different perspectives. Important tasks in this section include how to design equality, diversity and inclusion plans for a project, ensuring that equality, diversity and inclusion plans are implemented; training stakeholders about equality, diversity and inclusion.
g) **Training and Capacity Development:** The tasks in this specific area include supporting applicants in developing impactful research proposals, supporting the development and strengthening of partnerships to ensure research impact is realised at a local level; monitor ongoing impact and how it relates to the overall project goals; supporting further development of impacts whether that be through additional funding, dissemination, or reporting.
h) **Impact and Sustainability:** Tasks include ensuring training and capacity development activities are clearly defined throughout the application; support and maintaining collaborative partnerships to encourage training; support training activities and capacity building activities; support knowledge exchange activities and dissemination at the end of a project.
i) **Ethical, Legal and Social Implications:** RMAs often have to ensure thorough ethics reviews prior to application submissions or projects starting; ensuring contracts are in place detailing all obligations; assisting researchers to demonstrate an awareness of the social and ethical implications of their research and supporting the collection and storage of data and feeding back findings to research participants.

The scoping tool was piloted and then fine-tuned before it was implemented. The average completion time of the scooping to was 15 minutes. The target population for the scoping exercise were RMAs in Africa and in the UK and the scoping tool was distributed broadly via a press-release provided in both French and English. The scoping tool was also disseminated via various mailing lists of the participating universities, research consortia, RMA societies such as ARMA. Descriptive and inferential analyses of the results of the scoping exercise were conducted in STATA. Thematic analyses were conducted on free text responses. Ethical approval was obtained from the Human Research Ethics Committee of the Faculty of Health Sciences, University of Cape Town (R023/2015). All participants provided written consent.

## Results

### Characteristics of participants

 A total of 108 participants completed the scoping tool with 49% from Africa countries (n=49) and the rest from the UK (n=59). Respondents from Africa came from 15 countries (Algeria 1, Cameroon 1, Cote d’ Ivoire 1, Ghana 2, Kenya 1, Malawi 1, Mali 1, Nigeria 4, Somalia 1, South Africa 23, Uganda 1, Sudan 1, Tanzania 2, Zambia 1, Zimbabwe 8). There was a significant difference in the gender distribution of the participants from the UK and African countries, with African countries having twice as many males as the UK. The 55–64 age was more common in African participants (12%) against 2% (p=0.055) in the UK.

A statistically significant number of participants were employed in the academic sector (P-value 0.001) in comparison to those employed by government, private and research institutes (see Table 2). The UK (71%) had statistically significantly more permanent staff members among participants compared to Africa (39%), thus, African participants were more contract-based. There was a high preponderance of African participants being employed as Research Managers/Coordinators (n=61%), whereas in the UK the highest proportion of respondents were employed as Finance/Contract officers (59%) and research administrators. There is a statistically significant difference between the number of participants in the Finance/Contracts and other roles between the Africa and the United Kingdom. Significantly more Finance/Contract officers in the UK compared to Africa (p=0.001), and more Research Managers/Coordinators in Africa than in the UK (p=0.0132). Other roles mentioned by participants which were provided as free text included: Postdoctoral fellow; Lecturer; Innovation hub manager; Bioanalytical chemist; Junior scientist; Bioanalytical laboratory technician; Laboratory scientist; Research project administrator; Founding director; Co-Director doctoral training centre; Ethics committee secretariat; Knowledge transfer; Health and safety; Risk assessment; Conflict resolution; Strategy development; Advocacy; Pre-award and post-award administration; GDPR Data guidance; Supporting research through laboratory analyses and sample processing in clinical trials; Due diligence; Pastoral care; Research proposal development; Strategic research and impact delivery and reporting; Marketing and comms; Running calls for funding; Supporting industrial partnerships and research/training opportunities; Creating and delivering large events; Contributing to and delivering the vision of the project; Consultant; Chancellor and chief scientific officer; Deputy Dean 60% of responds in the UK had more than three years of experience in the RMA field while only 35% of participants from Africa had more than 3 years ( Table 3).

**Table 2.** Characteristics of participants who responded to the scoping tool.

Category	Variable	Africa	UK	P-value
**Age (Years)**	<25	1 (0.02)	0 (0)	
	25–34	16(0.33)	11 (01.9)	
	35–44	18 (0.38)	19 (0.32)	
	45–54	11 (0.23)	22 (0.37)	
	55–64	1 (0.02)	7 (0.12)	
	65–75	1 (0.02)	0 (0)	
	>75	0	0	
**Gender**	Female	31 (0.65)	47 (0.80)	0.037
	Male	17 (0.35)	10 (0.17)	
	Other	0	2 (0.03)	
**Location**	Continent	45%	55%	--
**Type of employer**	Academic	31 (0.63)	57 (0.97)	
	Research Institute	16 (0.33)	1 (0.015)	
	Private Companies & Government Department	2 (0.04)	1 (0.015)	
**Highest level of completed qualification**	Self-taught	0	0	
	Informal training by peers	1	0	
	Certified short course certificate	2	0	
	Diploma	2	3	
	Undergraduate degree	6	12	
	Postgraduate diploma	3	1	
	Honours	2	4	
	Masters	12	12	
	PhD	21	28	
	Other	0	2	
**Training specific for your current role** ** you have received**				
	Self-taught	22 (0.45)	36 (0.61)	0.094
	Informal training by peers	20 (0.41)	44 (0.75)	0.0004
	Certified short course	13 (0.27)	10 (0.17)	0.225
	Diploma	2 (0.04)	3 (0.05)	0.805
	Undergraduate degree	6 (0.12)	1 (0.02)	0.027
	Postgraduate Diploma	4 (0.08)	2 (0.03)	0.281
	Honours	2 (0.04)	2 (0.03)	ns
	Masters	8 (0.16)	5 (0.08)	0.211
	PhD	12 (0.24)	10 (0.17)	0.332
	Other	3 (0.06)	8 (0.14)	
**Employment Status Between the UK and African participants**				
Type of Role	Finance/Contract Officer	18% (9)	59% (33)	0.001
	Research Man/Coordinator	61% (30)	37% (32)	0.013
	Principal Investigator	16% (8)	17% (10)	0.931
	Other	26.5% (13)	7% (4)	0.005
Permanent Versus Contract/Research	Permanent	39% (19)	71 (42)	0.0007
	Contract/Research based	59 (30)	29 (17)	0.0015

**Table 3.** Roles versus job description.

Area		Finance/Contract	Project Managers/ Coordinators	Principal Investigators
Type of role	Leadership	23	50	84
	Management	53	72	47
	Operational	77	80	63
Areas in which tasks are performed	Equitable partnership	0.37	0.44	0.74
	Finance Management	0.58	0.59	0.42
	Project Management	0.46	0.85	0.74
	Monitoring and Evaluation	0.28	0.55	0.37
	Reporting and Communications	0.46	0.76	0.84
	Equity, Diversity & Inclusion	0.12	0.35	0.52
	Training and Capacity Development	0.39	0.72	0.85
	Impact and Sustainability	0.23	0.52	0.74
	Ethical, Social, Legal and Social Implications	0.3	0.53	0.68

**Figure 1.**
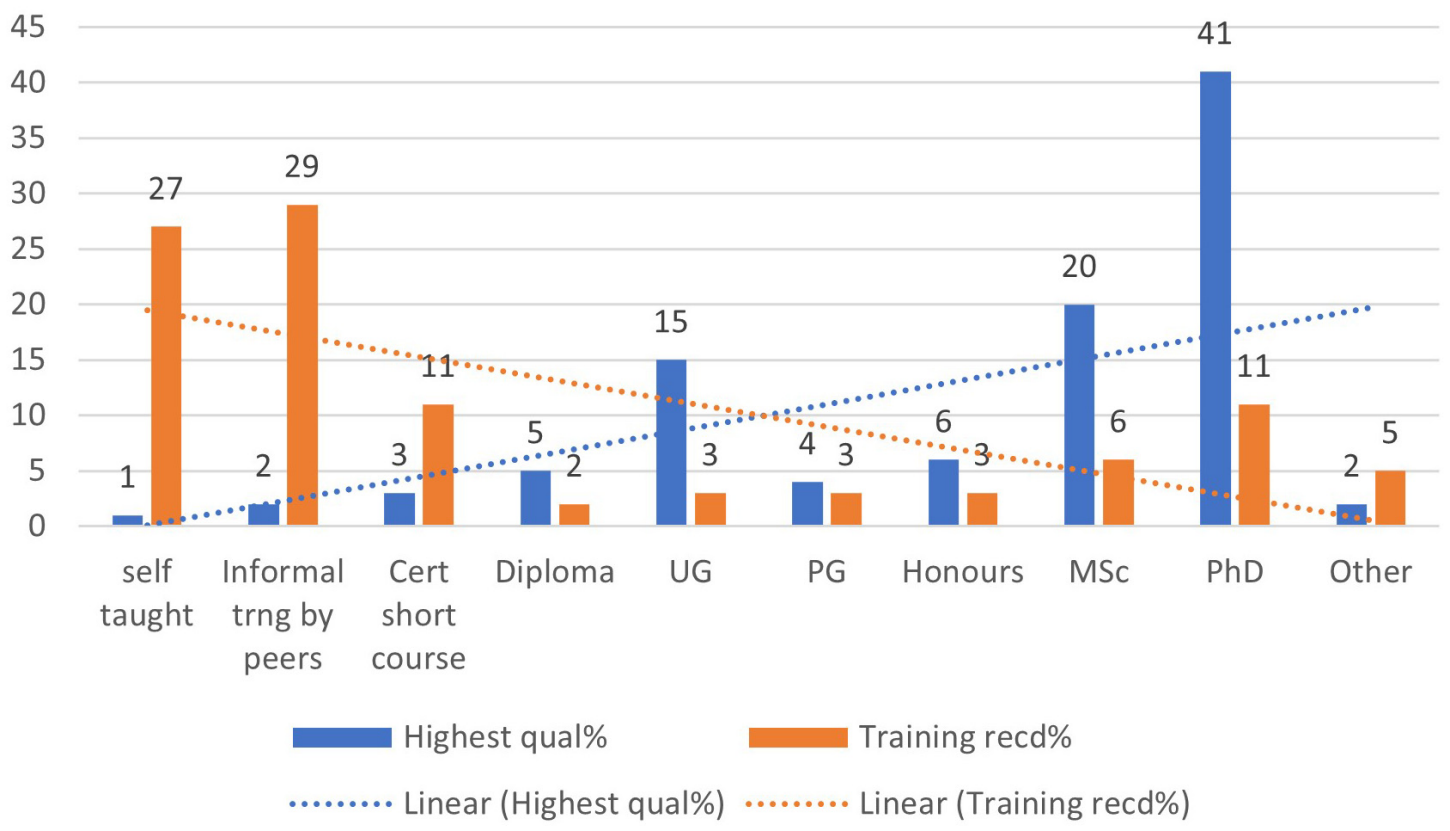
Inverse relationship between highest qualification versus training for current job.

***Highest qualification versus training for the current job.*** The formal qualifications: PhD, MSc and Undergraduate, were the highest attained in this order. As illustrated in Table 2, there was no statistically significant difference in the distribution of highest qualification between African and UK participants. Most of the RMAs received training for their current post from informal peer training followed by self-teaching and certified short courses. Of note is that there is an inverse relationship between the highest qualification attained versus training for the current job ( Figure 1).

***Roles versus job description.*** Most of the respondents irrespective of their location had an operational, management and leadership roles. In addition, all the participants irrespective of their job description worked across all the identified competency areas. There was not significant difference between the UK versus Africa. Irrespective of participants’ job categories’ they reported conducting tasks in all the competency areas identified in the Method section, see Table 3 for details.

### Confidence

The participant’s confidence levels in performing several tasks differed between the UK and African participants as illustrated in the heatmap shown in Table 4. We considered confidence levels of the groups to be high-level if more than 70% of the participants selected “strongly confident and confident”. The high-level confidence areas are highlighted in green. Table 4b shows a Heatmap of confidence levels against competency areas (rounded to the nearest 10). Highlighted are areas where there are more >20% differences in responses between African and European participants. There were differences between African and the UK participants (Africa versus UK, respectively), with respect to confidence in promoting or supporting mutual learning (90% versus 40%), lack of confidence in procurement (20 and 50%), confidence in project management platforms (60% versus 40%), coordination and communication (90% versus 60%), Designing of M&E frameworks and indicators (60% versus 30%) , conducting data analyses (70% versus 40%) and engaging with media (50% versus 20%), respectively. These results could be a reflection of the differences in the composition of the participants from the UK versus Africa.

**Table 4.** Heatmap of Confidence levels against competency areas.

	Africa- SC+C	UK- SC+C	Africa- NT	UK- NT	Africa- NC+NSC	UK- NC+NSC
Building trust within partnership	83	75	12	15	18	17
Facilitating shared goals, responsibilities and resources	88	79	6	17	6	4
Navigating funding obligations	50	62	25	21	25	17
Contributing to a fair working environment	29	13	47	48	12	13
Promoting or supporting mutual learning	94	39	0	43	6	17
Reporting or evaluation taking account differing needs of target audiences	60	54	27	33	13	12
Developing budgets	64	81	14	19	23	0
Ensuring adherence to funders terms and conditions	64	77	14	15	23	8
Procurement	47	29	33	19	19	52
Monitoring budget, tracking expenditure or cashflow forecasting	61	56	9	17	30	26
Processing financial claims or overseas/cross-border payments	52	50	13	14	34	37
Audit trails, reporting to funders and or management boards	45	50	14	18	41	32
Preparation of bids between international partners	46	71	27	15	26	15
Establishing project plans or policies	70	70	17	22	13	8
Project management platforms	62	26	26	37	13	37
Coordination and communication	93	59	3	30	3	11
Achieving project deliverables	91	69	3	23	6	8
Building or maintaining relationships with funders, partners or other stakeholders	87	75	7	25	7	0
Collecting and Collating data	86	68	7	19	7	14
Designing of M&E frameworks and indicators	63	28	11	44	26	28
Conducting due diligence of partners or sub-awardees	53	44	26	17	21	39
Collecting monitoring data	66	50	32	33	0	17
Conducting data analyses	65	39	20	33	15	28
Writing evaluation reports	67	55	19	22	15	23
Designing communications plan	55	44	29	32	16	20
Implementing communications plans	68	64	19	20	13	16
Engaging with the media	48	17	32	30	19	52
Developing funder reports	69	88	12	4	18	8
Responsible for engaging with key stakeholders	72	57	9	27	19	42

**Table 4b.** Heatmap of Confidence levels against competency areas (rounded to the nearest 10). Highlighted are areas where there are more >20% differences in responses between African and European participants.

	Africa- SC+C	UK- SC+C	Africa- NC+NSC	UK- NC+NSC
Building trust within partnership	80	80	20	20
Facilitating shared goals, responsibilities and resources	90	80	10	0
Navigating funding obligations	50	60	30	20
Contributing to a fair working environment	30	10	10	10
Promoting or supporting mutual learning	90	40	10	20
Reporting or evaluation taking account differing needs of target audiences	60	50	10	10
Developing budgets	60	80	20	0
Ensuring adherence to funders terms and conditions	60	80	20	10
Procurement	50	30	20	50
Monitoring budget, tracking expenditure or cashflow forecasting	60	60	30	30
Processing financial claims or overseas/cross-border payments	50	50	30	40
Audit trails, reporting to funders and or management boards	50	50	40	30
Preparation of bids between international partners	50	70	30	20
Establishing project plans or policies	70	70	10	10
Project management platforms	60	30	10	40
Coordination and communication	90	60	0	10
Achieving project deliverables	90	70	10	10
Building or maintaining relationships with funders, partners or other stakeholders	90	80	10	0
Collecting and Collating data	90	70	10	10
Designing of M&E frameworks and indicators	60	30	30	30
Conducting due diligence of partners or sub-awardees	50	40	20	40
Collecting monitoring data	70	50	0	20
Conducting data analyses	70	40	20	30
Writing evaluation reports	70	60	20	20
Designing communications plan	60	40	20	20
Implementing communications plans	70	60	10	20
Engaging with the media	50	20	20	50
Developing funder reports	70	90	20	10
Responsible for engaging with key stakeholders	70	60	20	40

### Challenges versus enablers for previous successful relationships

**Figure 2.**
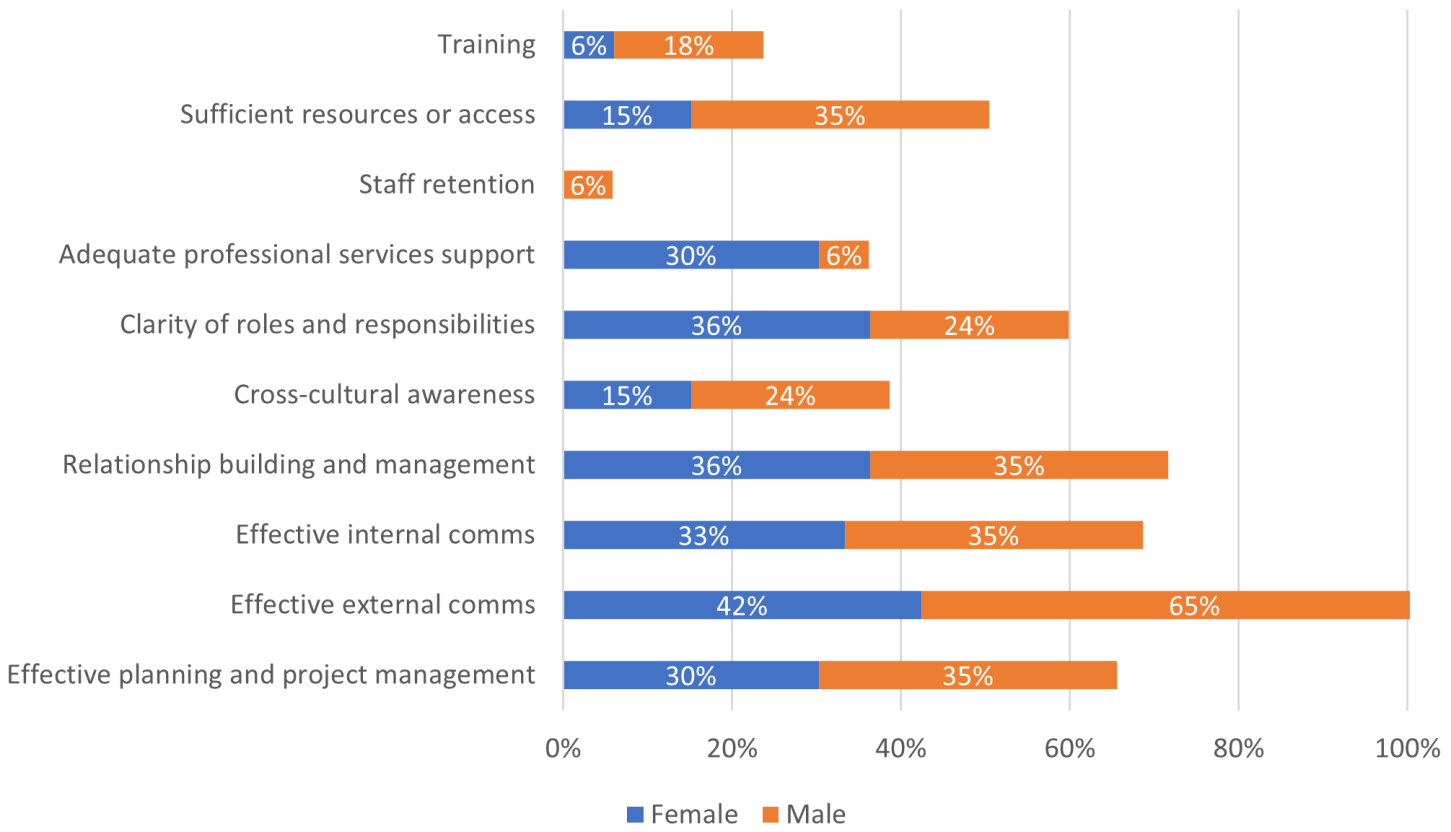
Approaches identified by respondents that helped previous collaborative projects to be successful. Results recorded as percentage of all respondents identified as male or female.

All the challenges that were provided in the scoping tool were selected by the participants. Only a few qualitative challenges were reported. Issues covered cross-cultural and cross-sector understanding (“differences in definitions across cultures/sectors”), adequate planning (“Insufficient time spent on preparation.”) and poor external and internal systems (“Financial oversight has not been streamlined”). Participants were asked to provide free text responses on what made previous collaborations a success, a thematic analysis was conducted, and several themes emerged. Effective communication followed by effective planning and project management emerged as key facilitators for successful collaborations as shown in Figure 2. The challenges seem to map onto the “what has worked well” questions, i.e. the “what has worked well responses” cited approaches that would overcome most of the challenges listed by participants (see Table 5a and Table 5b).

**Table 5a.** Sample quotations on “What has worked well?”.

Role type	Country	Experience	What worked well in previous collaborative projects?
Manager/Coordinator & Principal Investigator	South Africa	7–9 years	*When a team has good a work ethic; individuals assigned and dedicated to a role;* * accountability*
Manager/Coordinator & Principal Investigator	South Africa	> 12 years	*Regular, fixed project meetings; clear deliverables for each team member;* * administrative grant support*
Administrator	South Africa	> 12 years	*Excellent communication; Transparency between stakeholders; Good relations*
Research Support	United Kingdom	10–12 years	*A shared understanding of requirements from both sides*
Manager/Coordinator	South Africa	4–6 years	*Consistent and clear communication with collaborators. Be knowledgeable about* * timelines which will help with practical implementation of project. Learn about the* * culture and local context*
Research Support	United Kingdom	1–3 years	*Open communication from start of project BID with all partners and internal support* * roles. Clear, simple and agreed budgets with all partners. Good organisation skills to* * know what deadlines or milestones are approaching and communicating this with * *relevant partners and roles*
Manager/Coordinator	United Kingdom	> 12 years	*Champion staff within different departments (especially within Finance and IT) who* * have helped to 'unblock' and resolve issues/requests that have got stuck in a system* *as it does not follow business as usual (particularly with overseas projects)*
Principal Investigator	Somalia	> 12 years	*Clear and constant communication; transparency; and resources*
Research Support	United Kingdom	4–6 years	*Good relationships between the administrative functions who help to deliver*
Research Support	United Kingdom	> 12 years	*1) Maintain key staff in critical posts for as much of the project lifetime as possible; * *2) Clarify and document roles, responsibilities & expectations amongst partners* * at the outset (even though they are likely to change through the project lifetime) * *building trust & empathy; 3) Plan, agree, and document a 'win-win' collaboration * *as it is inequities (considering areas like resources, cultural contexts, and sharing of* *benefits/outputs) and mismatched or misunderstood expectations that can damage * *partnerships*
Research Support	United Kingdom	> 12 years	*In my experience, the most effective collaborative projects are those that arise from* * long-established working relationships with international partners, so each has good * *familiarity with the research strengths, challenges and needs of the others.*
Manager/Coordinator	United Kingdom	4–6 years	*1) Pump-priming - Projects which start small (in terms of partnership and funding* * amounts) generally have time to develop capacity and capability so are well placed* * to scale up and manage larger programmes. 2) Flexible internal approaches - where* * decision making has allowed for non-standardised processes to be followed, which * *are often better suited for international partnerships 3) Dedicate administrative * *resource to support project delivery*
Manager/Coordinator	Ghana	7–9 years	*Anticipating expenditure and providing funds in advance reduces delay in executing* * project objectives. Also, making required forms for accountability available with * *specified timelines reduces the strain involved in reporting and serves as a guideline* * in following standard protocols.*
Manager/Coordinator	United Kingdom	10–12 years	*Regular communication, established long term relationships where trust is built up * *over time. Knowledge of how foreign payments system works and how to work within* * its constraints*
Manager/Coordinator	South Africa	7–9 years	*Getting to know the partners better as people. Getting to understand some of their * *challenges.*

**Table 5b.** Sample quotations on challenges.

Role type	Experience	Highest qual.	Training received for role	Country	Challenges encountered by respondents
Manager/Coordinator & Principal Investigator	7–9 years	PhD	Certified short course	South Africa	*Financial oversight has not been* * streamlined and automated, this causes* * enormous delays for transfer of sub * *awardee funds.*
Manager/Coordinator	>12 years	Masters	Self-taught / informal training by peers	United Kingdom	*Time-zone issues / differences in* * expectations /differences in definitions* * across cultures and sectors.*
Principal Investigator	>12 years	PhD	PhD	Somalia	*Procuring instruments and chemicals. Also* * sending samples outside the country.*
Research Support	>12 years	PhD	Self-taught / informal training by peers / Certified short course	United Kingdom	*Insufficient time spent on preparation* * / roles / and responsibilities / financial * *obligations and rules.*
Manager/Coordinator	7–9 years	PhD	Self-taught / informal training by peers / Certified short course	Uganda	*Poor internet connections and language* * barriers.*
Manager/Coordinator	7–9 years	PhD	Self-taught / informal training by peers / Masters	South Africa	*Different work ethics and different cultures.*

### Resources

Participants were asked to highlight resources or skills they are willing to share with others in the future. A number or participants indicated their willingness to share skills and knowledge. The SMARTlife team also compiled a list of resources which could be of benefit to RMAs new to the field. These resources have been categorized according to the competence areas identified in this work and are freely accessible from the SickleInAfrica ( Makani *et al.*, 2020) website ( SMARTLife, 2021a).

## Discussion

Currently, the RMA profession is generally ill-defined, poorly understood and therefore is barely recognized in some developing countries. A number of publications confirm the need for RMA professionals for the preparation and implementation of effective research projects ( Langley & Green, 2009; Tauginienė, 2009; Virágh *et al*., 2019). The growing trend towards increased collaborative multi-institute and international research projects further emphasize the need to recognize, strengthen and professionalize the RMA field. Documenting baselines and needs of target populations is a recommended approach for strengthening implementation plans. This report is one of a handful of scoping exercises conducted in the RMA field and provides insights into the current status quo of RMAs’ training and roles in the UK and Africa and has potential to be useful in informing collaborative projects and training programmes for RMAs.

The need for formal training programmes is highlighted by results from this scoping exercise and this is not a new finding ( Langley & Green, 2009; Tauginienė, 2009). Most of the participants are self-taught and their formal qualification is not aligned to their current job role. While professional organizations such as ARMA offer training opportunities for their members, membership are quite steep. There is need to augment the ongoing association-based training opportunities with formal university-based qualifications. Unfortunately, most of the training available for RMAs are available and were developed in developed countries such as the United States of America and the UK and might not cater effectively for the needs of RMAs in different regions. A note-worthy achievement for Africa was the establishment of MSc programme which was recently established at the University of Stellenbosch ( Langley & Barsby, 2020). However, there is need to provide short term certification such as post graduate short courses and diplomas which might be fit for purpose taking into account the fact that most current RMAs already have PhDs and MSCs. There is a challenge to provide courses in lower-resourced research environments which are affordable to the RMA community. Ongoing work within institutions and the sector to raise the profile of RMAs and acknowledge the contribution they make towards a successful research ecosystem is one way to advocate for increased resources for formalised RMA training.

If not fully understood, differences in the profiles and experiences of professionals in a collaborative project may limit the success of any research project. Lower job security in African participants versus the UK counterparts could lead to less continuity and ineffective partnerships if the risk of personnel changes is not mitigated upfront during the project planning. While some of the differences are risks and required mitigation, some differences in participants’ profiles could be leveraged for capacity strengthening and mutual learning. For example, the increased years of experience highlighted by the UK counterparts could be leveraged to strengthen specific skills in the lesser experienced collaborators.

Skills and competency-based assessments have potential to inform training and project partnerships and such assessments have been used in a range of fields including bioinformatics, genomics and other management professionals. An added advantage is that a set of competency areas and specific tasks can also be used in longitudinal assessments to inform adjustments and optimisation of research partnerships as well as for the development and adjustments of training programmes. While this study highlights differences between the African and UK participants in confidence levels of specific areas, we acknowledge while these differences could be reflecting reality, these differences could also simply be a reflection of the differences in the job roles of the participants who took part in this scoping review and this require further exploration.

## Conclusions

A qualitative study is recommended to probe further some of the results observed from this work. A more thorough literature review is recommended in the future, for example to compile a more exhaustive list of existing competence areas. After the scoping exercise had been concluded, we discovered we had omitted the supporting and sourcing of institutional and project infrastructure as a key competence area. The roles of RMAs in supporting project infrastructure has been highlighted in the eighteen parameter Higher Education Institutional Capacity Assessment Tool (HEICAT) which was developed by the International Research Exchanges Board (IREX) to gauge performance of academic institutes ( HEICAT, 2019).

This work has potential to inform the development of more formal competence-based courses or programs to teach RMA as a profession. The cost of the courses needs to be affordable to RMAs. We also recommend stability in the contracts for RMA posts in-order to retain expertise. The RMAs highlighted the broadness of their roles, therefore streamlining of duties for RMAs could improve their efficiencies. In addition to informing training and the working conditions for RMAs, results from this work highlight differences between RMAs that may be useful for informing and promoting equitable international collaborations.

## Data availability

Figshare. SmartLife Infographic. DOI: https://doi.org/10.6084/m9.figshare.17097164.v1

Figshare. SmartLife Data Dictionary. DOI: https://doi.org/10.6084/m9.figshare.17099798.v2

Data are available under the terms of the Creative Commons Zero "No rights reserved" data waiver (CC BY 4.0 Public domain dedication).
