# Supplementary material for: Competency-based Training Needs Assessment for Research Managers and Administrators in Africa and the United Kingdom to Strengthen Equitable Partnerships
Source: AAS Open Res. 2022 Mar 10;5:11. [Version 1] doi: 10.12688/aasopenres.13272.1 (PMC9648359; doi:10.12688/aasopenres.13272.1)
Supplement: Peer review history

# Peer review: Reviewer response for version 1

**DOI:** 10.21956/aasopenres.14395.r29281

**Published:** 2022-08-08

**Authors:** Carolynn Thomas Jones

**Affiliations:** 1 Center for Clinical and Translational Science, The Ohio State University, Columbus, OH, USA

This is an excellent article that highlights the needs for Research Managers and Administrators for grants and contracts training.  The differences between UK and Africa participants are somewhat predictable due to the differences in roles; however there is an increasing need, even amongst academic institutions in the US for improved training for research administration training. There is a professional association the writers may benefit from reviewing - srainternational.org that could be another source for training activities, some are free, others may have costs ($99US).  

 In addition to the Joint Task Force for Clinical Trial Competencies (https://mrctcenter.org/clinical-trial-competency/) there is definitely a need for better project management and research administration training. The SMARTLIfe Competencies would be a wonderful addition to clinical research education.

Is the work clearly and accurately presented and does it cite the current literature?

Yes

If applicable, is the statistical analysis and its interpretation appropriate?

Yes

Are all the source data underlying the results available to ensure full reproducibility?

Yes

Is the study design appropriate and is the work technically sound?

Yes

Are the conclusions drawn adequately supported by the results?

Yes

Are sufficient details of methods and analysis provided to allow replication by others?

Yes

Reviewer Expertise:

I have > 30 yrs experience in clinical research management and education. I was one of the key researchers in the PERC study, providing capacity building in global clinical research  (Wilson et al.: https://doi.org/10.1002/chp.21167) where one component was teaching how to train and then measuring local outreach training.

I confirm that I have read this submission and believe that I have an appropriate level of expertise to confirm that it is of an acceptable scientific standard.

# Peer review: Reviewer response for version 1

**DOI:** 10.21956/aasopenres.14395.r29206

**Published:** 2022-06-06

**Authors:** Rakeshnie Ramoutar-Prieschl

**Affiliations:** 1 Department of Research and Innovation, University of Pretoria, Pretoria, South Africa

Nembaware *et al. *have shared a much needed and timely research undertaking that addresses the scarce skills areas of Research Managers and Administrators (RMAs) and clearly outlines the need for trained RMAs. The literature review is well laid out. The SMARTLife project, as proposed in this study, uses a set of competencies to capture existing roles as well as training needs and gaps through a comparative assessment of participants from the United Kingdom and Africa. Three main areas were identified: (i) Demographic profile; (ii) Levels of confidence in specific competence areas; and (iii) Aspects related to future collaborations with SMARTLife. These three (3) sections were further broken down into nine competency areas: equitable partnerships; financial management; project management; monitoring & evaluation; reporting & communications; equity, diversity & inclusion; training & capacity development; impact & sustainability; and ethical, legal & social implications. These are well detailed in the article. The research questions are well articulated, and the methodological approach is feasible, based on the study imperatives. The results, discussion and conclusion provide for much needed food for thought and provides baseline information that has the potential to inform more formal competence-based courses, modules and programmes for RMA training and development.

 Suggested points for consideration:

1. There are some typographical errors e.g. In the introduction section: (i) RMAS vs RMAs; (ii) Table 1: line 1, last column: “Not for profit *&”* organizations ; (iii) Table 3: last row: “Ethical, *Social*, Legal and *Social* Implications”.
2. It would be worthwhile to include a comparison of the capacities and job roles of RMAs as well as the number and size of projects across the institutions in the UK and Africa.
3. Additional information on SARIMA: they have partnered with Stellenbosch University to develop a postgraduate diploma and masters programme on RMA. This may need to be included in Table 1. However, despite such efforts, affordable and accessible courses are very much needed, as stated by the authors.
4. It would be worthwhile to consider the following references:
  1. **Building Science Systems in Africa**** ***Edited *** by Hanlin, R., Tigabu, A.D., and Sheikheldin, G. (2021) ****accessible at: http://www.acts-net.org/publications/books/building-science-systems-in-africa - specific reference to the chapter on “ *Building Sustainable Research Management Capacity in Africa”*
  2. **Scaling up Professionalization of Research Management in Southern Africa **by Willianson, C., Dyason, K., and Jackson, J. (2020) accessible at: https://www.srainternational.org/blogs/srai-jra1/2020/05/03/scaling-up-professionalization-of-research-managem
    "The Professionalisation of Research Management project began at the 2016 INORMS Meeting. The project involved developing SARIMA’s strategy and a Professional Competency Framework (PCF), development of Guidelines for the International Professional Recognition Council and development of Guidelines for Professional Recognition of Research Managers. In 2018, the Guidelines for the International Professional Recognition Council and Guidelines for Professional Recognition of Research Managers in Southern Africa were finalized. Two categories of research professional were identified and given the following designations:
    Research Management Professional (RMP) and
    Senior Research Management Professional (SRMP)."

Is the work clearly and accurately presented and does it cite the current literature?

Yes

If applicable, is the statistical analysis and its interpretation appropriate?

Not applicable

Are all the source data underlying the results available to ensure full reproducibility?

No source data required

Is the study design appropriate and is the work technically sound?

Yes

Are the conclusions drawn adequately supported by the results?

Yes

Are sufficient details of methods and analysis provided to allow replication by others?

Yes

Reviewer Expertise:

Research management and policy

I confirm that I have read this submission and believe that I have an appropriate level of expertise to confirm that it is of an acceptable scientific standard.

# Peer review: Reviewer response for version 1

**DOI:** 10.21956/aasopenres.14395.r29168

**Published:** 2022-04-19

**Authors:** Golbahar Pahlavan

**Affiliations:** 1 European Clinical Research Infrastructure Network (ECRIN), Paris, France; 2 Council on Health Research for Development (COHRED), Geneva, Switzerland

Nembaware *et al. *clearly present the need for trained RMA, in particular in Africa, based on adequate literature review and a scoping survey addressed to UK and African research institutions. The scoping survey methodology uses competency areas from ARMA adjusted by the ad hoc SMARTLife project, bringing together RMAs from UK and Southern Africa, to develop a set of competencies and use the latter to conduct a needs assessment for international research collaborations comparing UK and Africa.

 The proposed three main sections of the scoping tool: demographic data, levels of confidence in specific areas, followed by the break-down into nine competency areas, are sound and thorough.

 The results are presented in tables and figures which summarize the survey findings including segmentation by gender and some qualitative results which enrich the data.

 The discussion and conclusion are well balanced and point to additional directions for providing a more thorough picture of RMA training needs.

 The article is timely and underscores the critical need for more quantity and quality RMA in Africa, outlining potential ways forward through training and institutional support.

 Suggested points for consideration:

1. Information regarding the portfolio of projects from the participation institutions e.g. number of running projects, timeline, funding sources and amount would provide additional objective parameters to better appreciate the need for RMA as well as the potential imbalance in resources and capacities between African institutions, as well as with the UK, which is not addressed in this paper.
2. It would be worthwhile to address in addition to the global competencies, the specific needs of African institutions to negotiate and manage research projects. The Research Fairness Initiative developed by the Council on Health Research for Development provides a balanced framework for setting up equitable partnerships including capacity building.
3. The tables and figures presented would benefit from a legend explaining the abbreviations used and the data presented.
4. The items covered in the Heatmaps of Confidence levels against competency areas Tables 4a and 4b would benefit from a classification/clustering based on the project life-cycle.

Is the work clearly and accurately presented and does it cite the current literature?

Yes

If applicable, is the statistical analysis and its interpretation appropriate?

Not applicable

Are all the source data underlying the results available to ensure full reproducibility?

No source data required

Is the study design appropriate and is the work technically sound?

Yes

Are the conclusions drawn adequately supported by the results?

Yes

Are sufficient details of methods and analysis provided to allow replication by others?

Yes

Reviewer Expertise:

Research management and policy-making

I confirm that I have read this submission and believe that I have an appropriate level of expertise to confirm that it is of an acceptable scientific standard, however I have significant reservations, as outlined above.

## References

[1] : Investing in health R&D: where we are, what limits us, and how to make progress in Africa. *BMJ Glob Health* .2019;4(2) : 10.1136/bmjgh-2018-001047 e001047 10.1136/bmjgh-2018-001047 30899571PMC6407556

[2] : “Get us partnerships!” - a qualitative study of Angolan and Mozambican health academics’ experiences with North/South partnerships. *Globalization and Health* .2020;16(1) : 10.1186/s12992-020-00562-7 10.1186/s12992-020-00562-7 32295611PMC7161017

[3] : Open Science in Kenya: Where are we?.2021; 10.31730/osf.io/mgkw3 10.31730/osf.io/mgkw3
